# Acute Ischemic Stroke in a Teenage Patient: Are We “MIS-C”ing Something?

**DOI:** 10.7759/cureus.47321

**Published:** 2023-10-19

**Authors:** Aleena Moin, Robert B Lowe, Biren J Desai

**Affiliations:** 1 Internal Medicine/Pediatrics, Geisinger Medical Center, Danville, USA; 2 Internal Medicine/Pediatrics, Geisinger Commonwealth School of Medicine, Scranton, USA

**Keywords:** pediatrics, multi-system inflammatory disease in children (mis-c), pediatric stroke, covid 19, pediatric neurology, acute ischemic stroke

## Abstract

The reported annual incidence of acute ischemic stroke (AIS) among pediatric and young adults is 1-13/100,000. In adults, ischemic stroke is attributed to several risk factors such as smoking, hypertension, atherosclerosis, and diabetes. Alternatively, pediatric ischemic stroke is associated with a broad spectrum of etiologies including prematurity, congenital heart disease, arteriopathies like moyamoya, chronic inflammatory disease, sickle cell, hypercoagulability, and malignancy. In rare cases, AIS has been associated with multisystem inflammatory syndrome in children (MIS-C), a Kawasaki-like inflammatory disease affecting patients younger than 21 years of age. This recently recognized and rare condition has been linked to severe acute respiratory syndrome coronavirus 2 (SARS-CoV-2) infection, and presentations can vary widely in terms of severity and systemic involvement. While the exact reason behind this association is unknown, there is a growing body of evidence in adult literature that links SARS-CoV-2 infection to hypercoagulability and immune-mediated thrombosis. In pediatric patients, this association is not very clear.

We report a case of a 17-year-old, previously healthy male who presented with acute-onset expressive aphasia, right-sided hemiparesis, and facial droop after two weeks from experiencing coronavirus disease 2019 (COVID-19)-like symptoms. A non-contrast head CT revealed an acute left M2 territory infarct while serum workup was consistent with MIS-C. Providers must maintain a high degree of suspicion and consider AIS in pediatric patients presenting with even mild neurological changes and a recent history of SARS-CoV-2 infection.

## Introduction

Acute ischemic stroke (AIS) is a rare phenomenon in the pediatric population. The majority of childhood strokes occur in the perinatal period while its reported incidence among pediatric and young adult patients is 1-13/100,000 per year [1]. In adults, ischemic stroke is associated with several well-established risk factors including smoking, hypertension, atherosclerosis, and diabetes. Alternatively, the pathogenesis of pediatric ischemic stroke is more commonly linked to a broad spectrum of etiologies including prematurity, congenital heart disease, arteriopathies like moyamoya, chronic inflammatory diseases, sickle cell syndrome, metabolic disease, hypercoagulability states, and malignancy [2].

Multisystem inflammatory syndrome in children (MIS-C) is a Kawasaki-like inflammatory disease affecting patients younger than 21 years of age. This newly recognized disease is a rare, but serious condition linked to severe acute respiratory syndrome coronavirus 2 (SARS-CoV-2) infection. Presentations of MIS-C vary widely in terms of severity and organ involvement. In rare cases, AIS has been associated with MIS-C [3]. We present a case of a young, previously healthy teenage male diagnosed with AIS in the setting of coronavirus disease 2019 (COVID-19) infection.

## Case presentation

**Figure 1:**
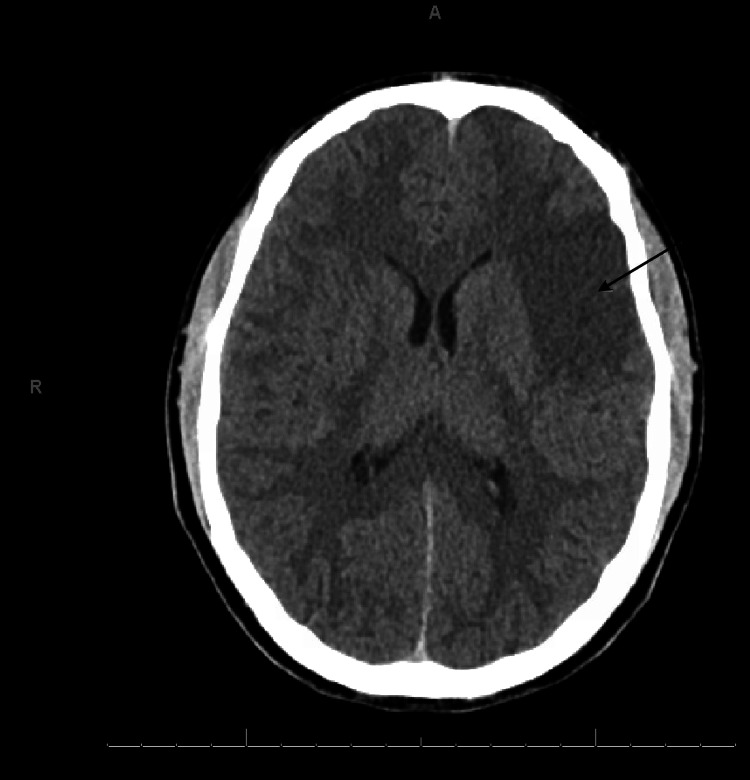
CT brain of the patient Arrow demonstrates left MCA-territory ischemic infarction CT: computed tomography; MCA: middle cerebral artery

A 17-year-old male presented to the emergency department with eight hours of expressive aphasia, right-sided hemiparesis, and right-sided facial droop. His National Institutes of Health Stroke Scale (NIHSS) score was documented as 9 upon presentation, signifying moderate impairment. A non-contrast head CT revealed an acute left M2 territory infarct (Figure 1).

**Figure 2:**
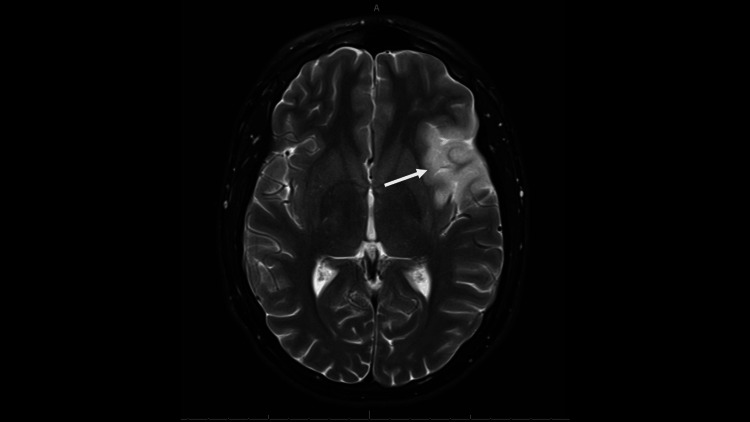
Axial, diffuse weighted image sequences from MRI of the brain Arrow demonstrates increased signal intensity, demonstrating evolving left MCA-territory ischemic infarction MCA: middle cerebral artery; MRI: magnetic resonance imaging

The patient was referred for mechanical thrombectomy, as he was outside the window for tissue plasminogen activator (tPA). Neurosurgery recommended a CT perfusion imaging study, which showed insufficient penumbra, and hence they recommended medical management only. An MRI obtained on hospital day two showed the evolution of his left M2 ischemic stroke (Figure 2).

As part of the AIS evaluation, an echocardiogram was also obtained, which revealed left ventricular (LV) apical hypokinesis, mildly reduced LV ejection fraction (45%), and an apical mural thrombus. The bubble study was negative for a patent foramen ovale (PFO). The patient was started on aspirin 81 mg and intravenous anticoagulation with heparin after consultation with pediatric cardiology and hematology.

He had no significant past medical or surgical history. The family history was negative for hypercoagulable conditions. A review of symptoms was positive for mild upper respiratory symptoms, fever, myalgia, odynophagia, abdominal pain, and diarrhea in the preceding two weeks. His pre-admission COVID-19 polymerase chain reaction (PCR) test was negative. However, numerous family members had confirmed COVID-19 infection, raising concerns for COVID-19-related MIS-C in the patient. Laboratory workup was significant for negative factor V Leiden mutation and anticardiolipin antibodies and demonstrated multisystem organ involvement, including elevated serum creatinine, cardiac enzymes, inflammatory markers, and positive SARS-CoV-2 antibody (Table 1) [4,5].

**Table 1:** Inflammatory and hypercoagulable workup results COVID-19: coronavirus disease 2019; SARS-CoV-2: severe acute respiratory syndrome coronavirus 2

	Result	Reference range
COVID-19 polymerase chain reaction (PCR)	Negative	Negative
Ferritin	3901 ng/mL	30-400 ng/ml
B-type natriuretic peptide (Pro-BNP)	12,405 pg/ml	<300 pg/ml
D-dimer	3.32 ug/m	<0.50 ug/m
Troponin	49 ng/L, 56 ng/L	<22 ng/L
C-reactive protein	218 mg/L	<5 mg/L
Erythrocyte sedimentation rate	31 mm/hr	<15 mm/hr
Serum creatinine	1.6 mg/dL	0.4-1.0 mg/dL
Albumin	2.9 g/dL	3.8-5.0 g/dL
SARS-CoV-2 antibody	Positive	Negative
Factor V Leiden	Negative	Negative
Anticardiolipin antibody panel	Negative	Negative

These findings, along with the reported history of fevers, confirmed the diagnosis of MIS-C based on the CDC and American Academy of Pediatrics (AAP) criteria. A multidisciplinary discussion with pediatric rheumatology, infectious disease, cardiology, and hematology led to the initiation of intravenous immunoglobulin (IVIG) (1 g/kg) two doses daily and IV methylprednisolone (30 mg/kg once then 1 mg/kg BID) along with continued heparin anticoagulation for the patient's LV thrombus. His inflammatory markers improved dramatically within 48 hours, and his neurological findings returned to near baseline. A repeat echocardiogram demonstrated a resolving LV thrombus. Anticoagulation was replaced with low-molecular-weight heparin (enoxaparin) and Xa levels were monitored to ensure therapeutic levels. Steroids were changed to oral prednisone 30 mg BID and tapered off over a three-week period. After 28 days of inpatient care, the patient was discharged to an inpatient acute rehab with plans to continue low-dose aspirin and low-molecular-weight heparin (0.5 mg/kg BID) for a total period of three months.

## Discussion

This case report highlights a unique presentation of SARS-CoV-2 infection resulting in a diagnosis of MIS-C. The association of AIS with MIS-C is rare [6], with only a few cases reported so far. In this case, widespread immune dysregulation resulted in acute shock-like syndrome, which led to global hypokinesis and LV thrombus formation with the subsequent cardioembolic phenomenon manifesting as left-sided M2 CVA.

Providers can refer to the WHO, CDC, and AAP diagnostic criteria if MIS-C is suspected. The treatment of MIS-C is primarily aimed at reducing inflammation and preventing further devastating outcomes related to this highly inflammatory state. As outlined in the MIS-C diagnostic criteria, it is imperative to exclude alternative diagnoses such as Kawasaki disease prior to starting therapy. A multidisciplinary discussion with infectious disease and rheumatology is recommended before administering immune-modulating therapies. For most hospitalized patients, the American College of Rheumatology recommends first-tier treatment with IVIG 2 g/kg per ideal body weight (IBW) given once, and high-dose methylprednisolone (1-2 mg/kg/d) tapered pending the patient's clinical response [7]. Our patient demonstrated dramatic improvement after treatment initiation, with the normalization of inflammatory markers and resolution of neurologic deficits.

This case report also highlights the lack of consensus guidelines for the treatment of AIS in children. This can be attributed to several factors. As mentioned, pediatric strokes are far less common than adult strokes and are very heterogeneous in prevalence, etiology, and clinical presentation. The prevalence is highest in the perinatal period, with numerous etiologies varying dramatically by age, and clinical presentation varies widely. There has been a paucity of research on the safety and efficacy of well-established adult modalities in the treatment of pediatric stroke [8]. Emerging evidence suggests that both endovascular thrombectomy and tPA, such as tenecteplase, may be viable treatment options for pediatric patients [9,10]. However, further studies with larger numbers of participants are required before their use can be established as standard practice. Our patient was evaluated for mechanical thrombectomy but was not a suitable candidate due to a lack of penumbra. Fortunately, his neurovascular deficits drastically improved with the treatment of his MIS-C.

## Conclusions

The pathology of acute stroke in pediatric and young adult patients is very different than in adults. There is a growing body of evidence in adult literature that links SARS-CoV-2 infection to hypercoagulability and immune-mediated thrombosis. In pediatric patients, this association is not very clear. Providers should maintain a high index of suspicion and consider MIS-C in pediatric patients presenting with acute neurological changes and a recent history of SARS-CoV-2 infection.
